# Clinico-Angiographic Profile of Paramilitary Forces Personnel Presenting With Acute Coronary Syndrome at a Tertiary Care Institute in Jammu and Kashmir, India

**DOI:** 10.7759/cureus.117308

**Published:** 2026-10-01

**Authors:** Sameer Purra, Aamir Rashid, Qayoom Yousuf, Imran Akram Khanday, Imran Hafeez, Hilal Rather

**Affiliations:** 1 Department of Cardiology, Cityheart Superspeciality Hospital, Barsar, IND; 2 Department of Cardiology, Sher-i-Kashmir Institute of Medical Sciences, Srinagar, IND

**Keywords:** acute coronary syndrome, armed forces personnel, cardiovascular risk factors, coronary artery disease, myocardial infarction, percutaneous coronary intervention

## Abstract

Background: Acute coronary syndrome (ACS) is a leading cause of morbidity and mortality in middle-aged adults. However, there is a lack of data on its clinical profile, cardiovascular risk factors, angiographic characteristics, management, and outcomes in armed forces personnel. Thus, this study was conducted to evaluate the clinical characteristics, risk factors, angiographic profile, treatment strategies, complications, and outcomes of ACS among paramilitary forces personnel.

Methods: This study included 173 paramilitary forces personnel diagnosed with ACS. Demographic and service-related characteristics, cardiovascular risk factors, clinical presentation, Killip class, laboratory and echocardiographic parameters, time to revascularization, coronary angiographic findings, treatment strategies, procedural complications, and clinical outcomes were evaluated.

Results: The mean age of participants was 46.5±7.52 years, and 98.8% (n=171) were male. The most frequent cardiovascular risk factors were smoking (n=99, 57.2%) and hypertension (n=94, 54.3%), followed by dyslipidemia (n=58, 33.5%) and diabetes mellitus (n=49, 28.3%). Anterior wall myocardial infarction was the most common presentation (n=74, 42.8%), followed by inferior wall myocardial infarction (n=64, 36.99%) and non-ST-elevation myocardial infarction (n=21, 12.1%). The mean ejection fraction was 45.6±7.49%, and regional wall motion abnormalities were seen in 76.9% (n=133) of patients. Pharmaco-invasive therapy was the preferred treatment strategy in 68.8% (n=119), with thrombolysis being done in 72.3% (n=125). Coronary angiography was performed in 88.4% (n=153) of cases. The most common angiographic finding was single-vessel disease (n=82, 53.6%), and the left anterior descending artery was the most frequently involved vessel (n=92, 61.7%). The majority (n=99, 78.0%) of patients who underwent percutaneous coronary intervention (PCI) had a single stent. The most common electrical complications were complete heart block (n=8, 4.6%) and ventricular tachycardia (n=7, 4.0%).

Conclusions: ACS in paramilitary forces personnel predominantly affected middle-aged men and was associated with a high prevalence of modifiable cardiovascular risk factors, especially smoking and hypertension. Main management strategies were pharmaco-invasive therapy and coronary intervention. Early identification of cardiovascular risk factors and prompt reperfusion may improve prognosis in this population.

## Introduction

Cardiovascular diseases (CVDs) are still the number one cause of death worldwide, with an estimated 19.8 million deaths in 2022, with coronary heart disease (CAD) being a large component [1]. The Coronary Artery Disease in the Young (CADY) registry, enrolling 997 young patients from 22 Indian centers, documented acute coronary syndrome (ACS) in 68% of subjects and showed a considerable prevalence of traditional cardiovascular risk factors like hypertension, diabetes, smoking/tobacco use, and family history of CAD [2]. The burden of CAD is especially important in low- and middle-income countries like India, where it is often seen at a younger age [3].

ACS, encompassing ST-segment elevation myocardial infarction (STEMI), non-STEMI (NSTEMI), and unstable angina, continues to be a significant cause of cardiovascular morbidity and mortality. Clinical and angiographic characteristics may vary by age, geographic region, and underlying risk-factor profile. In a study of young patients with acute myocardial infarction, Smoking was the most common risk factor, whereas single-vessel disease and left anterior descending artery involvement were the most common angiographic findings [4,5]. Similarly, the Srinagar Percutaneous Coronary Intervention (PCI) Registry provided useful regional data about the clinical and angiographic profile of patients undergoing PCI in Kashmir [6].

Paramilitary forces represent a unique occupational group in which cardiovascular risk may be modified by both traditional risk factors and occupational circumstances. Paramilitary personnel may experience long-term operational demands, psychological stress, irregular schedules, and difficult working conditions. Importantly, cardiovascular risk factors are not lacking in otherwise physically fit military populations. In a large cohort of deployed military personnel with ACS, McGraw et al. reported a high prevalence of cardiovascular risk factors, including smoking, hyperlipidemia, and hypertension [7]. Additionally, a study of patients undergoing coronary angiography in an Indonesian military population observed differences in the severity of angiographic disease according to military rank, with lower Gensini scores among high-ranking personnel compared with low-ranking personnel and civilians [8].

These factors are especially important for paramilitary personnel stationed in Jammu and Kashmir, where their operational responsibilities may involve challenging terrain, hard labor, and inconsistent access to sophisticated cardiac care. Delays between the start of symptoms, hospital presentation, reperfusion, and coronary revascularization might affect myocardial salvage and clinical outcomes, making prompt diagnosis and treatment of ACS crucial. However, there is still a dearth of information regarding the clinical presentation, cardiovascular risk-factor profile, echocardiographic results, coronary angiographic features, reperfusion patterns, and in-hospital outcomes of paramilitary forces personnel in Jammu and Kashmir, India, who present with ACS. Thus, the aim of this study was to describe the clinical profile, cardiovascular risk factors, angiographic findings, treatment strategies, and in-hospital outcomes of ACS in paramilitary personnel admitted to Sher-i-Kashmir Institute of Medical Sciences (SKIMS) and 92 Base Hospital in Srinagar, Jammu and Kashmir, India. Secondary objectives were to describe time to reperfusion and echocardiographic findings. 

## Materials and methods

This was an ambispective study conducted at the Department of Cardiology of SKIMS and 92 Base Hospital, located in Srinagar, Jammu and Kashmir, India. The time period for collecting data retrospectively was July 2022 to April 2023, while a prospective study was conducted from May 2024 to July 2024. The study was conducted after approval from the Institutional Ethics Committee of SKIMS (approval number: SIMS 131/IEC-SKIMS/2023-350, dated September 20, 2023). Written informed consent was obtained from all prospectively enrolled participants or their legally authorized representatives, as applicable.

Study population

Eligibility Criteria

All paramilitary forces personnel admitted with ACS, including STEMI, NSTEMI, and unstable angina (UA) during the study period were eligible. Inclusion and exclusion criteria are given in Table 1. 

**Table 1:** Inclusion and exclusion criteria

Category	Criterion
Inclusion	Paramilitary forces personnel admitted with a diagnosis of acute coronary syndrome (ACS).
	Patients presenting with ST-segment elevation myocardial Infarction (STEMI), Non-ST-segment eevation myocardial Infarction (NSTEMI), or unstable angina.
	Patients who provided written informed consent for participation.
Exclusion	Patients presenting with chronic stable angina.
	Patients with severe renal insufficiency, defined as creatinine clearance <30 mL/min.
	Patients who did not provide informed consent.

Sample Size

The convenience sampling method was used, and a total of 173 patients were included in the study. Patients whose relevant clinical, laboratory, echocardiographic, angiographic, treatment, and outcome data were available from hospital records were included retrospectively, while eligible patients admitted during the prospective component of the study period were enrolled prospectively following informed consent. The retrospective and prospective components were analyzed together as a single cohort.

Definitions

ACS was diagnosed according to the Fourth Universal Definition of Myocardial Infarction [9]. Myocardial infarction was defined by evidence of myocardial injury with a rise and/or fall in cardiac troponin in the appropriate clinical setting, together with evidence of myocardial ischemia. STEMI was diagnosed on the basis of characteristic ischemic symptoms and persistent ST-segment elevation in the appropriate contiguous leads, in accordance with the diagnostic framework applicable during the study period. NSTEMI was diagnosed in patients fulfilling the criteria for acute myocardial infarction without persistent ST-segment elevation.

Smoking was defined as regular consumption of >1 cigarette/day for >6 months or cessation within the preceding two years. Diabetes mellitus was defined by fasting plasma glucose ≥126 mg/dL, HbA1c ≥6.5%, random plasma glucose ≥200 mg/dL in the presence of compatible symptoms, or current use of antidiabetic medication. Hypertension was defined as a previously documented diagnosis of hypertension and/or current treatment with antihypertensive medication. Dyslipidemia was defined according to established lipid thresholds or current lipid-lowering therapy. Significant alcohol consumption was defined according to established sex-specific thresholds.

Clinical assessment

A detailed history was obtained regarding symptom onset, hospital presentation, reperfusion treatment, and time to reperfusion. Demographic characteristics, cardiovascular risk factors (smoking, hypertension, diabetes mellitus, dyslipidemia, and alcohol use), family history of myocardial infarction, and substance use were recorded. Elevated blood pressure measurements obtained during the acute ACS presentation were not considered sufficient by themselves to establish a diagnosis of chronic hypertension. Clinical examination included assessment for heart failure, abnormal heart sounds, pericardial involvement, arrhythmias, and other complications of acute myocardial infarction.

Significant disease (CAD) was defined as ≥70% stenosis in a major epicardial coronary artery or ≥50% stenosis of the left main coronary artery. Procedural success was defined as residual stenosis <10% following stent implantation or <50% following balloon angioplasty, with thrombolysis in myocardial infarction (TIMI) grade 3 flow and absence of major angiographic or in-hospital clinical complications.

Killip classification

The Killip Classification for Heart Failure is a bedside clinical tool, originally published by Killip and Kimball in 1967, that segregates patients according to the severity of their post-myocardial infarction heart failure [10]. Killip class I patients were those without heart failure. Killip class II had mild heart failure with rales involving one third or less of the posterior lung field and systolic blood pressure of 90 mm Hg or higher. Killip class III had pulmonary edema with rales involving more than one third of the lung field and systolic blood pressure of 90 mm Hg or higher. Killip class IV were those in cardiogenic shock with any rales and systolic blood pressure less than 90 mm Hg [4]. 

Reperfusion strategy

STEMI patients presenting within 12 hours of symptom onset were assessed for reperfusion. Primary PCI was performed when feasible. Patients without contraindications to fibrinolysis who could not undergo timely primary PCI underwent fibrinolysis. Rescue PCI was performed following failed thrombolysis, whereas a pharmaco-invasive strategy was used after successful fibrinolysis according to clinical and logistical considerations. Door-to-needle time, door-to-balloon time, and total ischemic time were recorded. Time to revascularization was defined as the interval from symptom onset to the first definitive coronary revascularization procedure among patients who underwent revascularization [11]. Patients managed conservatively were excluded from this analysis.

Coronary Angiography and PCI

Coronary angiography was performed to assess the location, severity, morphology, and extent of CAD. Patients were categorized as having single-, double-, or triple-vessel disease according to the number of major coronary vessels with significant stenosis. Lesion characteristics, including lesion type (A, B, or C), ostial location, bifurcation, calcification, eccentricity, tortuosity, thrombus, chronic total occlusion, and coronary segment involved, were documented according to the American College of Cardiology (ACC)/American Heart Association (AHA) lesion classification and established angiographic criteria [12].

Procedural details included vascular access, number and location of lesions treated, stent characteristics, adjunctive pharmacotherapy/devices, and procedural complications. TIMI flow grade was recorded before and after intervention.

Investigations and echocardiography

Baseline investigations included complete blood count, blood glucose, renal function tests, lipid profile, and cardiac biomarkers. Transthoracic echocardiography was performed at baseline to assess left ventricular systolic function, regional wall-motion abnormalities, cardiac dimensions, and mechanical complications. 

Clinical and procedural outcomes

The study assessed procedural complications, including death, periprocedural myocardial infarction, stroke, emergency coronary artery bypass grafting, vascular complications, bleeding, coronary perforation, stent thrombosis, flow-limiting dissection, side-branch loss, significant arrhythmias, and contrast-associated acute kidney injury. Target vessel failure, target lesion failure, target vessel revascularization, target vessel myocardial infarction, cardiac death, and in-stent restenosis were recorded according to contemporary standardized definitions. Procedural death was defined as death attributed to a complication of the index invasive/procedural treatment, whereas deaths not attributed to the procedure were classified as non-procedural.

Statistical analysis

Data were entered into Microsoft Excel (Microsoft Corporation, Redmond, Washington, United States) and analyzed using IBM SPSS Statistics for Windows, version 20.0 (IBM Corp., Armonk, New York, United States). Statistical analysis was primarily descriptive because the study was designed to characterize the clinical, angiographic, treatment, and outcome profile of ACS in armed forces personnel. Continuous variables were expressed as mean ± standard deviation (SD), while categorical variables were presented as frequencies and percentages. No prespecified inferential subgroup comparisons were performed; therefore, no hypothesis-testing p-values or effect estimates are reported. The findings are interpreted as descriptive observations within the study cohort.

## Results

Out of 173 patients included in the study, 106 (61.3%) were retrospectively enrolled, and 67 (38.7%) were prospectively included (Table 2).

**Table 2:** Distribution of study subjects (N=173)

Study subjects	Frequency	Percentage
Prospective	67	38.7
Retrospective	106	61.3

Among the 173 study participants, the mean age was 46.5±7.52 years (range, 32-59 years), with most participants aged 41-50 years (n=69; 39.9%). The cohort was predominantly male (n=171; 98.8%), belonged mainly to the Central Reserve Police Force (CRPF) (n=92; 53.2%), were non-commissioned personnel (n=143; 82.7%), and were engaged in field duties (n=126; 72.8%). Previous high-altitude deployment was reported by 18.5% (n=32), as shown in Table 3.

**Table 3:** Sociodemographic and service characteristics of study cohort (N=173)

Parameter	Sub-category	Frequency (Percentage)
Age group (years)	31–40	45 (26.0)
	41–50	69 (39.9)
	51–60	59 (34.1)
Sex	Male	171 (98.8)
	Female	2 (1.2)
Regiment	Central Reserve Police Force (CRPF)	92 (53.2)
	Border Security Force (BSF)	37 (21.4)
	Regular Army	10 (5.8)
	Other forces	34 (19.6)
Rank type	Commissioned	30 (17.3)
	Non-commissioned	143 (82.7)
Job type	Field	126 (72.8)
	Desk	47 (27.2)
High-altitude deployment	Yes	32 (18.5)
	No	141 (81.5)

Smoking was the most prevalent cardiovascular risk factor (n=99; 57.2%), followed by hypertension (n=94; 54.3%), dyslipidemia (n=58; 33.5%), type 2 diabetes mellitus (n=49; 28.3%), significant alcohol consumption (n=23; 13.3%), and family history of CAD (n=17; 9.8%), as shown in Table 4.

**Table 4:** Cardiovascular risk factors (N=173)

Risk factor	Frequency (Percentage)
Smoking	99 (57.2)
Hypertension	94 (54.3)
Dyslipidemia	58 (33.5)
Type 2 diabetes mellitus	49 (28.3)
Significant alcohol consumption	23 (13.3)
Family history of coronary artery cisease	17 (9.8)

The most common clinical presentation was anterior wall myocardial infarction (AWMI) in 74 (42.8%), followed by inferior wall myocardial infarction (IWMI) in 64 (36.99%). NSTEMI was observed in 21 (12.1%), while high lateral wall myocardial infarction (HLWMI) and unstable angina were each reported in seven (4.0%) patients. Regarding Killip class, 72 (41.6%) patients were in Class II, followed by 63 (36.4%) in Class I, 31 (17.9%) in Class III, and seven (4.0%) in Class IV. The mean time to revascularization among the 145 patients who underwent primary PCI, rescue PCI, or a pharmaco-invasive strategy was 8.9±7.86 hours. The mean transport time was 10.5±8.39 hours, with 83 (48.0%) patients having a transport time of 5-10 hours (Table 5).

**Table 5:** Clinical presentation and initial severity Data are presented as frequencies (percentages), except for Time to revascularization and Transport time, which are also presented as mean±SD. Time to revascularization was analyzed among 145 patients who underwent primary PCI, rescue PCI, or a pharmaco-invasive strategy. AWMI: anterior wall myocardial infarction; IWMI: inferior wall myocardial infarction; HLWMI: high lateral wall myocardial infarction; NSTEMI: non-ST-elevation myocardial infarction; PCI: percutaneous coronary intervention

Parameters		Frequency (Percentage)
Clinical presentation	AWMI	74 (42.8)
	IWMI	64 (36.99)
	HLWMI	7 (4.0)
	NSTEMI	21 (12.1)
	Unstable angina	7 (4.0)
Killip class	I	63 (36.4)
	II	72 (41.6)
	III	31 (17.9)
	IV	7 (4.0)
, Time to revascularization (n=145)	Mean ± SD	8.9 ± 7.86
	<5 hours	43 (29.7)
	5–10 hours	66 (45.5)
	10–15 hours	24 (16.6)
	≥15 hours	12 (8.3)
Transport time	Mean ± SD	10.5 ± 8.39
	<5 hours	21 (12.1)
	5–10 hours	83 (48.0)
	10–15 hours	43 (24.9)
	≥15 hours	26 (15.0)

Among the 173 patients, the mean hemoglobin (Hb) was 13.9 ± 1.57 g/dL, total leukocyte count (TLC) was 9.97 ± 3.25 ×10³/µL, and neutrophil-to-lymphocyte ratio (NLR) was 5.04 ± 2.71. The mean platelet count was 200.5 ± 80.83 ×10³/µL, urea 38.9 ± 17.58 mg/dL, and creatinine 0.96 ± 0.28 mg/dL. Mean blood sugar was 122.3 ± 38.89 mg/dL, HbA1c 6.37 ± 1.49%, low-density lipoprotein (LDL) 131.1 ± 35.51 mg/dL, high-density lipoprotein (HDL) 37.2 ± 9.48 mg/dL, and total cholesterol 213.1 ± 46.87 mg/dL. The mean ejection fraction (EF) was 45.6 ± 7.49% (Table 6).

**Table 6:** Laboratory findings

Parameter	Mean ± SD
Hemoglobin (g/dL)	13.9 ± 1.57
Total Leukocyte Count (×10³/µL)	9.97 ± 3.25
Neutrophil-to-Lymphocyte Ratio	5.04 ± 2.71
Platelet Count (×10³/µL)	200.5 ± 80.83
Urea (mg/dL)	38.9 ± 17.58
Creatinine (mg/dL)	0.96 ± 0.282
Blood Sugar (mg/dL)	122.3 ± 38.89
Glycated Hemoglobin (%)	6.37 ± 1.49
Low-Density Lipoprotein (mg/dL)	131.1 ± 35.51
High-Density Lipoprotein (mg/dL)	37.2 ± 9.48
Total Cholesterol (mg/dL)	213.1 ± 46.87
Ejection Fraction (%)	45.6 ± 7.49

On echocardiography, regional wall motion abnormality (RWMA) was present in 133 (76.9%) patients. Mild and moderate mitral regurgitation (MR) were observed in 61 (35.3%) and 27 (15.6%) patients, respectively. Ventricular septal rupture (VSR) was noted in three (1.7%), free wall rupture (FWR) in one (0.6%), and pericardial effusion in nine (5.2%) patients. Pump failure was present in 31 (17.9%), while right ventricular (RV) failure was observed in five (2.9%). Grade 1, Grade 2, and Grade 3 diastolic dysfunction were present in 50 (28.9%), 75 (43.4%), and 12 (6.9%) patients, respectively (Table 7).

**Table 7:** Echocardiographic parameters

Echocardiographic finding	Frequency (Percentage)
Regional Wall Motion Abnormality	133 (76.9)
Mild Mitral Regurgitation	61 (35.3)
Moderate Mitral Regurgitation	27 (15.6)
Ventricular Septal Rupture	3 (1.7)
Free Wall Rupture	1 (0.6)
Pericardial Effusion	9 (5.2)
Pump Failure	31 (17.9)
Right Ventricular Failure	5 (2.9)
Grade 1 Diastolic Dysfunction	50 (28.9)
Grade 2 Diastolic Dysfunction	75 (43.4)
Grade 3 Diastolic Dysfunction	12 (6.9)

Thrombolysis was performed in 125 (72.3%) patients, with pharmacoinvasive therapy being the predominant intervention (n=119, 68.8%). Tenecteplase was the most frequently used thrombolytic agent (n=49, 39.2%). Coronary angiography was performed in 153 (88.4%) patients, with femoral access used in 94 (61.4%), as shown in Table 8.

**Table 8:** Revascularization and interventional management

Variable		Frequency (Percentage)
Mode of intervention	Primary Percutaneous Coronary Intervention	20 (11.6)
	Rescue Percutaneous Coronary Intervention	6 (3.5)
	Pharmacoinvasive	119 (68.8)
	Conservative	28 (16.2)
Initial management strategy	Thrombolysis	125 (72.3)
	Primary Percutaneous Coronary Intervention	20 (11.6)
	Referral for Coronary Artery Bypass Grafting	12 (6.9)
Thrombolytic agent (n=125)	Tenecteplase	49 (39.2)
	Streptokinase	45 (36.0)
	Reteplase	31 (24.8)
Coronary angiography	Performed	153 (88.4)
	Not performed	20 (11.6)
Coronary angiography access (n=153)	Radial	59 (38.6)
	Femoral	94 (61.4)

Of the 153 patients who underwent coronary angiography, four had normal coronary angiographic findings and were therefore excluded from vessel-specific and lesion-level analyses; consequently, these analyses included 149 patients with angiographically identifiable CAD. Single-vessel disease was the predominant angiographic pattern (n=80, 53.6%), and the left anterior descending artery (LAD) was the most frequently involved coronary artery (n=92, 61.7%). Type A lesions constituted 50.3% (n=75) of lesions. Among the 145 patients undergoing PCI, 127 underwent coronary stent implantation, and a single stent was used in 78.0% (n=99) of cases (Table 9).

**Table 9:** Coronary angiographic and PCI findings ACC: American College of Cardiology; AHA: American Heart Association; PCI: percutaneous coronary intervention

Parameters		Frequency (Percentage)
Vessel disease (n=153)	Single-Vessel Disease	82 (53.6)
	Double-Vessel Disease	51 (33.3)
	Triple-Vessel Disease/ Left Main–Triple-Vessel Disease	16 (10.5)
	Normal angiography	4 (2.6)
Coronary artery involved (n=149)	Left Anterior Descending Artery	92 (61.7)
	Right Coronary Artery	57 (38.3)
	Left Circumflex Artery	49 (32.9)
	Diagonal	13 (8.7)
	Left Main Coronary Artery	10 (6.7)
	Obtuse Marginal Artery	5 (3.4)
ACC/AHA lesion type (n=149)	Type A	75 (50.3)
	Type B	46 (30.9)
	Type C	28 (18.8)
Number of stents (n=127)	One	99 (78.0)
	Two	26 (20.5)
	Three	2 (1.6)

Electrical complications included complete heart block in eight patients (4.6%), ventricular tachycardia in seven (4.0%), AIVR in four (2.3%), and ventricular fibrillation in three (1.7%). Overall, 13 (7.5%) deaths were reported. Stent thrombosis, side-branch occlusion, no-reflow, and periprocedural myocardial infarction were among the documented procedural complications (Table 10).

**Table 10:** Complications, adjunctive procedures, and outcomes

Parameters		Frequency (Percentage)
Electrical complications	Complete heart block	8 (4.6%)
	Ventricular Tachycardia	7 (4.0%)
	Accelerated Idioventricular Rhythm	4 (2.3%)
	Ventricular Fibrillation	3 (1.7%)
Additional interventional modalities	Glycoprotein IIb/IIIa inhibitors	60 (34.7%)
	Intravascular Ultrasound	27 (15.6%)
	Plain Old Balloon Angioplasty	7 (4.0%)
	Drug-Eluting Balloon	2 (1.2%)
	Optical Coherence Tomography	3 (1.7%)
	Fractional Flow Reserve	3 (1.7%)
Major outcomes/complications	Deaths	13 (7.5%)
	Procedural deaths	10 (5.8%)
	Non-procedural deaths	3 (1.7%)
	Pump failure	5 (2.9%)
	Refractory arrhythmia	2 (1.2%)
	Ventricular Septal Rupture/ Free Wall Rupture	3 (1.7%)
	Side-branch occlusion	3 (1.7%)
	Stent thrombosis	2 (1.2%)
	No-reflow	1 (0.6%)
	Periprocedural Myocardial Infarction (MI)	1 (0.6%)
	Stroke	1 (0.6%)
	Contrast-Induced Nephropathy (CIN)	1 (0.6%)

## Discussion

Premature CAD is becoming more common in developing countries like India because to the epidemiological shift, which frequently affects people in their prime working years [2]. Military and paramilitary personnel represent an occupational population in which traditional cardiovascular risk factors may coexist with demanding operational and occupational circumstances. The present study describes the cardiovascular risk factors and clinical profile of ACS in paramilitary personnel stationed in Jammu and Kashmir, India.

With a mean age of 46.5 years, the study population was predominantly male (n=171; 98.8%). The gender makeup of the paramilitary forces is reflected in the male predominance. Our results are consistent with those of McGraw et al., who observed that all subjects in deployed United States military personnel with ACS were male and had a mean age of 44.7 years [7]. Despite having a younger mean age (35.8 years), Qadir et al. reported a similar male preponderance (89.47%) in young Kashmiri AMI patients [4]. While the military personnel in our cohort were older than that seen in Qadir et al.'s study [4], they were still younger than general population CAD cohorts in studies such as that conducted by Beig et al., who reported a mean age of 59.3 years [6]. Male predominance is expected in military populations and mirrors other Indian studies that studied army personnel [13].

Most of the patients were non-commissioned officers (n=143; 82.7%), with CRPF and Border Security Force (BSF) personnel forming the largest subgroups. Field-based roles and high-altitude deployments characterize the stressful and physically demanding environments faced by this population. Suryawan et al. demonstrated that low-ranking military personnel had significantly higher Gensini scores, indicative of more severe coronary artery disease, possibly due to heightened operational stress [8]. 

The two biggest risk factors were found to be smoking (n=99; 57.2%) and hypertension (n=94; 54.3%). In a study of serving Indian Army personnel, Srivastava et al. reported prehypertension in 63.0%, hypercholesterolemia in 22.0%, and current smoking in 19.2% of participants [13]. According to McGraw et al., 28% of United States soldiers had hypertension, and 47% smoked [7]. Even higher rates of smoking (79.8%) and hypertension (74.8%) were observed by Beig et al. in a civilian Kashmiri cohort, indicating a significant impact of regional lifestyle characteristics [6].

AWMI was the most common clinical presentation, occurring in 74 patients (42.8%), followed by IWMI in 64 patients (36.99%). This is similar to the findings of Qadir et al., who found that 47.3% of young adults had AWMI [4]. Similarly, according to Beig et al., 33.6% of patients had AWMI [6]; LAD involvement is a defining characteristic in these populations, as evidenced by the regularity of AWMI prevalence.

Revascularization took an average of 8.9 hours, while hospital transport took an average of 10.5 hours. These prolonged intervals likely reflect the distinctive referral and transport challenges associated with geographically remote postings and difficult terrain in the study setting. Because the present study did not formally determine the causes of delay, these findings should be interpreted as observed system-level intervals rather than being attributed to specific operational factors.

Pharmacoinvasive therapy was the predominant reperfusion strategy in the present cohort, being used in 68.8% (n=119) of patients. Such an approach is clinically relevant when timely primary PCI cannot be achieved. Contemporary STEMI guidelines recommend fibrinolysis when PCI-mediated reperfusion cannot be achieved within the recommended time window, followed by transfer to a PCI-capable centre and routine early angiography after successful fibrinolysis [11,14,15].

Tenecteplase and streptokinase were frequently employed in thrombolysis (n=125, 72.3%) of patients. While Beig et al. discovered that primary or elective PCI was the most common method (61.4%) [6], Qadir et al. reported a 68.4% thrombolysis rate in young adults [4]. Differences from other cohorts may reflect differences in patient characteristics, referral pathways, treatment availability, institutional practice, or study populations.

The mean left ventricular EF in our cohort was 45.6 ± 7.49%, while regional wall-motion abnormalities were present in 76.9% of patients. Mitral regurgitation, pericardial effusion, pump failure, and occasional mechanical complications were also documented. Qadir et al. similarly evaluated left ventricular systolic function in young patients with acute myocardial infarction; however, direct comparison of the additional structural and mechanical complications was avoided because these outcomes were not comprehensively reported in that study [4]. LAD was the most frequently affected artery (n=92, 61.7%), while single-vessel disease predominated (n=82, 53.6%). Similar findings were reported by Beig et al. [6] and Qadir et al. [4], although the reported frequencies differed between studies. The present cohort demonstrated an adverse cardiometabolic risk profile, with a mean LDL cholesterol of 131.1±35.51 mg/dL, mean HDL cholesterol of 37.2±9.48 mg/dL, and mean HbA1c of 6.37±1.49%. These findings reinforce the importance of systematic identification and control of modifiable cardiovascular risk factors among serving military personnel [6,13].

In our analysis, type A lesions were the most common lesion morphology (n=75, 50.3%), followed by type B (30.9%) and type C lesions (18.8%), which is in line with the 52.3% (type A lesions) reported by Beig et al. [6]. These findings describe the angiographic lesion morphology observed in the present cohort and should not be interpreted as a direct measure of overall disease severity. The frequency of high-grade stenosis and lesion complexity may provide additional information regarding angiographic disease burden, but causal explanations related to occupational stress or delayed detection cannot be established from the present descriptive study.

Rare but clinically important complications included pump failure and mechanical complications such as ventricular septal rupture. In this study, 13 (7.5%) deaths were reported. The observed mortality should be interpreted in the context of the study population, treatment patterns, and institutional setting, as the present descriptive study was not designed to determine the factors independently associated with mortality. In the study by Beig et al., the procedure fatality rate was very low [6]. 

Undiagnosed dyslipidemia and sedentary behavior among active troops were noted by McGraw et al. [16]. Suryawan et al. found that higher-ranking employees had lower Gensini scores, suggesting the protective effect of administrative positions and decreased stress exposure [8]. These findings are supported by our study and suggest differences in angiographic disease burden across occupational or rank groups; however, the present study did not perform risk-adjusted analyses by rank or occupational role.

Strengths and limitations

This study's focus on a distinct, high-risk population and thorough prospective and retrospective data collection are its strongest points. The lack of long-term follow-up and the underrepresentation of women are limitations that may affect how broadly the findings can be applied. Other limitations were convenience sampling, the ambispective design, potential information bias, limited generalizability, and the absence of risk-adjusted outcome analysis.

## Conclusions

In this ambispective cohort of paramilitary forces personnel presenting with ACS, the study population was predominantly middle-aged men with a high prevalence of smoking and hypertension. AWMI was the most frequent clinical presentation, while single-vessel coronary disease and LAD involvement were the predominant angiographic findings. Pharmacoinvasive therapy and thrombolysis were commonly used, reflecting the treatment patterns observed in this cohort. The findings provide descriptive regional data on the clinical and angiographic profile of ACS among armed forces personnel and support further evaluation of cardiovascular risk assessment and timely reperfusion strategies in this population.
